# Spectroscopic Analysis of Tryptophan as a Potential Optical Biomarker for Estimating the Time of Death

**DOI:** 10.3390/ijms252312915

**Published:** 2024-11-30

**Authors:** Emilia Gruszczyńska, Aneta Lewkowicz, Martyna Czarnomska, Joanna Koczur, Katarzyna Walczewska-Szewc, Michał Kaliszan, Łukasz Balwicki, Piotr Bojarski

**Affiliations:** 1 Institute of Experimental Physics, Faculty of Mathematics, Physics and Informatics, University of Gdansk, ul. Wita Stwosza 57, 80-308 Gdańsk, Poland; emilia.gruszczynska@phdstud.ug.edu.pl (E.G.); martyna.czarnomska@phdstud.ug.edu.pl (M.C.); piotr.bojarski@ug.edu.pl (P.B.); 2 Institute of Law Sciences, Faculty of Law and Administration, University of Silesia in Katowice, ul. Bankowa 11 B, 40-007 Katowice, Poland; joanna.koczur@us.edu.pl; 3 Institute of Physics, Faculty of Physics, Astronomy and Informatics, Nicolaus Copernicus University in Toruń, ul. Grudziądzka 5, 87-100 Toruń, Poland; kszewc@umk.pl; 4 Department of Forensic Medicine, Medical University of Gdańsk, ul. Dębowa 23, 80-204 Gdańsk, Poland; michal.kaliszan@gumed.edu.pl; 5 Department of Public Health and Social Medicine, Faculty of Health Science, Medical University of Gdańsk, ul. Dębinki 7, 80-211 Gdańsk, Poland

**Keywords:** tryptophan, molecular spectroscopy, time of death estimation, fluorescence, absorption, sweat–fatty substance, biomarker

## Abstract

The estimation of the time of death represents a highly complex and challenging task within the field of forensic medicine and science. It is essential to approach this matter with the utmost respect for human rights while acknowledging the inherent limitations of the current methods, which require continuous refinement and expansion. Forensic science recognizes the necessity to improve existing techniques and develop new, more accurate, and non-invasive procedures, such as physicochemical approaches, to enhance the precision and reliability of time of death determinations. This article proposes a novel, non-invasive method for estimating the time of death using a spectroscopic analysis of tryptophan. The initial phase of the study concerns the presentation of the spectroscopic properties of tryptophan at varying pH levels, with consideration given to the pH fluctuations that occur during the decomposition of cadavers. The findings confirm the stability of the spectroscopic properties at different environmental pH levels. Subsequently, preliminary trials were conducted on 15 healthy human volunteers, which demonstrated that tryptophan concentrations in fingerprint samples were within the detection limits using molecular spectroscopy techniques. The final objective was to ascertain whether the composition of the substance present on the skin surface of a deceased individual up to 48 h postmortem is comparable to that of the sweat–fatty substance in living individuals. This was confirmed by the absorption and emission spectral profiles, which showed overlapping patterns with those obtained from living volunteers. The most significant outcome at this stage was the demonstration of a considerable increase in emission intensity in the spectra for samples obtained approximately 48 h after death in comparison to that obtained from a sample taken approximately 24 h after death. This indicates a rise in the concentration of tryptophan on the skin surface as the postmortem interval (PMI) increases, which could serve as a basis for developing a tool to estimate the time of death.

## 1. Introduction

The determination of time of death (TOD) has constituted a significant challenge within the field of forensic medicine. Despite the considerable progress that has been made in this field and the development of numerous methods and techniques (including traditional, modern, and auxiliary approaches), the estimation of the time of death continues to present significant challenges. These challenges are primarily associated with the multitude of external influences that impede the predictable progression of postmortem changes. Consequently, the issue of accurately determining the time of death remains unresolved [1,2].

The estimation of the time of death in forensic medicine is based on the detection of characteristic changes, both chemical and physical, that occur after death. In this context, it is possible to distinguish between the early and late signs of death [3]. The early signs of death include the processes of algor mortis, rigor mortis, and livor mortis. The late post-mortem changes can be further divided into two categories: those of a decomposing nature (autolysis and putrefaction) and those of a perpetuating nature (fat–wax transformation, mummification, and mire transformation). One method for estimating the time since death (TSD) is to identify the stage of decay based on observations of the aforementioned changes [3].

At present, the precision of the TSD estimation is inadequate to fulfill the standards required in forensic medicine. Further research, encompassing both theoretical and experimental approaches, is required to enhance the precision of this estimation. Determining the time since death is aided by a comprehensive understanding of the circumstances surrounding the discovery of the body and the lesions present on it. Nevertheless, despite the fact that the course of some postmortem changes in the human body shows a certain dependence on the time since death, external factors can influence the process. These include ambient temperature, humidity, and the actions of parasites, insects, or animals. Furthermore, the corpse’s individual characteristics can also exert influences on the course of postmortem changes. Individual characteristics (weight, age, and sex) or internal factors (the actions of the corpse’s own enzymes or bacteria) can exert a significant influence on the course of postmortem changes, thereby making it challenging or even impossible to accurately interpret the results obtained [4]. Consequently, the determination of the time of death based on the observation of the classical signs of death should be classified as a typical method of a subjective nature, in which the experience of the forensic medical examiner also plays a role. Therefore, a further comprehensive experimental investigation is necessary to address this issue. It is evident that the accuracy of estimating the time of death can be enhanced by developing new markers and methodologies for this purpose, as well as by comparing various chemical and physical values that have already been identified.

The majority of the methodologies currently employed for this purpose rely heavily on the subjective assessment of postmortem changes within the human body that can be observed without the aid of instruments. As shown by the experiment conducted by Aydin in 2010 [5], the lack of definitive outcomes and discrepancies in the experiences of investigators significantly impact the final assessment. Moreover, the current methodologies possess several inherent disadvantages. Firstly, the use of professional equipment is required, which is not always available [6,7]. Secondly, the accuracy of the estimation of the time of death is significantly reduced with the passage of time since death. It is therefore necessary to propose a new, more objective method based on real data that will allow a more accurate estimation of the time of death. In recent years, new laboratory-based methods have emerged with the aim of improving the accuracy of the PMI determination [6,8,9]. These methods are based on the observation of biochemical alterations that occur rapidly following death in all bodily tissues, particularly in fluids such as blood, spinal fluid, and cerebrospinal fluid. The changes occur in a relatively orderly fashion until the body decomposes, but each change has its own time factor or rate. The identification of chemical alterations facilitates a more precise estimation of the interval since the occurrence of death. However, it should be noted that the biochemical profiles may vary depending on a number of factors, including preexisting conditions, the cause of death, survival, environmental factors, and the properties of the test substance. The most significant limitation of these enhanced methodologies is that they cannot be conducted at the scene. Transporting the body to an off-site facility where it will await examination could introduce additional variables that may affect the accuracy of the determination of the TSD [10,11].

The objective of our studies is to develop a novel methodology that will enable the collection of material in the field and a subsequent independent analysis, circumventing the necessity for an autopsy. The implementation of this methodology will facilitate the estimation of TSD values with greater speed and precision, thereby increasing the accuracy of the data, which is known to decrease significantly over time.

The employment of disparate methodologies for estimating the TOD cannot be adequately addressed without due consideration of human rights, particularly with respect to the dignity and humane treatment of human remains. It is thus imperative to guarantee that the proposed methodology is in compliance with human rights in this regard. The inherent dignity of the human being forms the basis of human rights and distinguishes humans from animals. This dignity is also extended to the deceased, as the human body remains an integral part of the person it once was and is deserving of respect even after death.

The concept of human dignity is of pivotal importance in modern ethics, bioethics, and human rights instruments.

Despite the absence of a universally accepted definition, it is frequently referenced. The Universal Declaration of Human Rights of 1948, for example, underscores the intrinsic dignity and equal rights of all individuals as the cornerstone of freedom, justice, and peace [12]. Similarly, the 1966 International Covenants on Civil and Political Rights and on Economic, Social, and Cultural Rights assert that these rights originate from the inherent dignity of the human person. Consequently, human dignity has become a fundamental tenet of modern social and international relations, influencing principles such as freedom of choice, integrity, and responsibility [13].

The European Bioethics Convention of 1997 and the EU Charter of Fundamental Rights of 2000 both place emphasis on the inviolability of human dignity and the necessity to respect and protect it [14,15]. Although the European Convention on Human Rights (1950) does not explicitly mention human dignity, it is implied as the foundation for all other human rights and freedoms [16].

The ethical principles that guide research involving human cadavers dictate that the deceased must be treated with respect. This is based on the conviction that human dignity is inextricably linked to the human body, even in the absence of life. The human corpse, which is inextricably linked to the person it once was, is deserving of respect. This notion is supported by the principle of pietism. This principle serves to safeguard the interests of both the deceased and their relatives, underscoring the necessity to respect their feelings. It is of the utmost importance to ensure the legal protection of the human body, both during life and after death, in order to maintain the dignity of the human being and the significance of human existence.

### New Potential Method

The potential of using amino acids to estimate the time of death was first reported in 1981 by Perry et al. [17], who demonstrated that the concentrations of specific amino acids in human brain tissue increased with an increasing postmortem interval (PMI). In subsequent years, independent research groups confirmed this finding by analyzing not only brain tissue but also cerebrospinal fluid, plasma, cisternal fluids, and vitreous humor [18,19,20,21]. The increase in the concentrations of free forms of amino acids in human PM tissues is related to the initial stage of body decomposition, autolysis. The phenomenon of autolysis, which occurs postmortem, is caused by the cessation of the oxygen supply and a reduction in cytoplasmic pH. This reduction disrupts cellular processes, including ion gradients and membrane integrity. Consequently, lysosomes within cells become destabilized, releasing their contents, including proteases, into the cytoplasm [22]. Proteases then catalyze the hydrolysis of peptide bonds in proteins, breaking them down into smaller peptide fragments. Subsequent enzymatic activity degrades these peptides into individual amino acids [23]. This process is enhanced after death by the acidic environment within cells resulting from the accumulation of metabolic by-products, such as lactic acid. Proteolysis occurs gradually and affects different tissues at varying rates [24].

A gradual elevation in the concentration of tryptophan was noted with a prolonged time since death. Tryptophan is an essential amino acid that belongs to the category of protein amino acids. Although some bacteria in the human microbiome are capable of producing tryptophan, the amino acid derived from this source does not significantly contribute to human physiology [25]. Tryptophan is primarily present in the blood and plasma in a bound state to the protein albumin, with only 10–20% of the total present in a free form. Tryptophan’s distinctive structural composition, based on the indole moiety, endows it with pronounced hydrophobic properties and facilitates the stabilization of proteins that contain it [26]. The spectroscopic properties of tryptophan are attributed to the indole ring, which allows it to absorb light radiation below 300 nm with a maximum at around 280 nm in aqueous solution. Upon excitation with light at the wavelength corresponding to the maximum absorption of tryptophan, it exhibits the ability to fluoresce. The range in which the emission spectrum of tryptophan in aqueous solution is observed is between 300 and 500 nm, with the emission maximum occurring at approximately 360 nm [27]. The potential of tryptophan as a biomarker for estimating TSD has already been demonstrated by Kärkelä, Grunblatt, and Ansari [20,28,29]. In their study, Kärkelä and Scheinin examined the concentrations of various metabolites, including tryptophan, in cisternal fluids following death. The results indicated a linear increase in tryptophan concentrations with increasing time after death [20]. Additionally, Grünblatt et al. demonstrated that, in contrast to the other compounds they analyzed, the tryptophan concentration increased in brain tissue with an increasing PMI. Moreover, the researchers demonstrated that age, gender, pathological states, and the agonal stage do not influence tryptophan levels [29]. In a separate study, Ansari and Menon employed tryptophan derived from the vitreous body as a means of estimating the time of death. The researchers employed the reagent o-phthalaldehyde (OPA), which exhibits a selective affinity for tryptophan, and allowed the detection level to increase. The findings of this research team also demonstrated the viability of utilizing tryptophan as a means of determining the time of death [28].

The spectroscopic properties of tryptophan are currently employed in a number of techniques, but the methodologies involved inevitably result in the compromise of the physical integrity of the remains. These methods typically entail the extraction of material from tissues that are challenging to access, such as the vitreous body of the eye or cerebrospinal fluid. In contrast, our proposed method represents a significant advancement in the field, as it enables the collection of samples from the surface of the deceased’s skin, thus maintaining the physical integrity of the body. This novel approach is non-invasive and, notably, obviates the necessity for transporting the corpse to a forensic department for sample collection. In contrast, the material can be conveniently and efficiently collected at the scene, thereby streamlining the process and preserving the condition of the remains.

## 2. Results and Discussion

The initial stage of the study comprised a spectroscopic analysis of tryptophan in solvents of varying pH, designed to simulate the conditions present within the human body at different stages of decomposition [30]. The objective of this study was to ascertain the stability of tryptophan in solution, with a particular consideration of the pH level and initial molar concentration, which is analogous to the concentration of tryptophan in human tissues postmortem.

**Figure 1:**
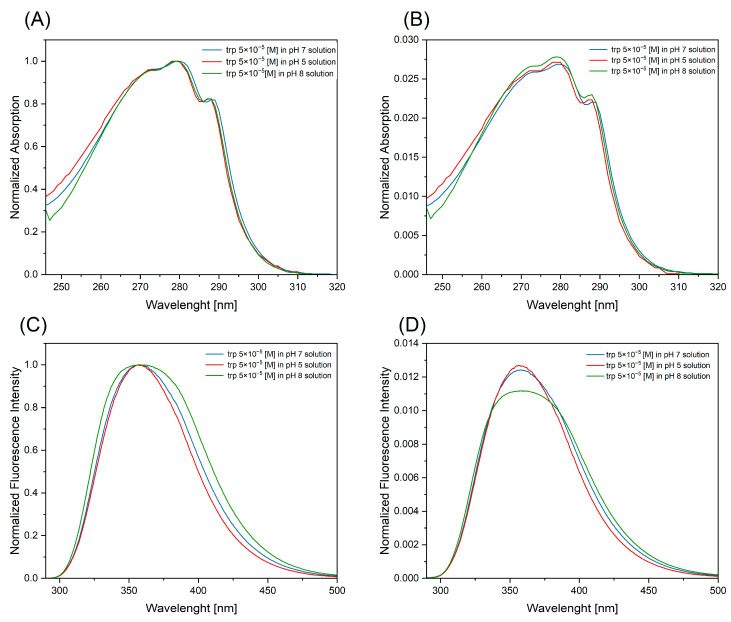
Spectroscopic spectra of reference tryptophan solutions with different pHs: (**A**) absorption spectra normalized to [0, 1]; (**B**) absorption spectra normalized to the area; (**C**) emission spectrum normalized to [0, 1] (excitation wavelength 280 nm); and (**D**) emission spectrum normalized to the area (excitation wavelength 280 nm).

Absorption measurements were conducted within the wavelength range of 245–310 nm. The optical contour of the absorption spectrum retained the shape characteristic of tryptophan, as observed in all of the analyzed solutions, with a maximum at a wavelength of 280 nm (Figure 1A,B). The fluorescence spectrum (Figure 1C,D) was obtained by the excitation of the samples with a wavelength of 280 nm, which was the absorption maximum for the solutions tested. This demonstrated the stability of tryptophan at the pHs considered. Minor discrepancies were noted at pH 8, and manifested as an expansion of the half-band width. In all cases, the fluorescence maximum was observed to be localized at approximately 360 nm. The fluorescence spectrum exhibited a range of wavelengths between 285 and 500 nm.

**Figure 2:**
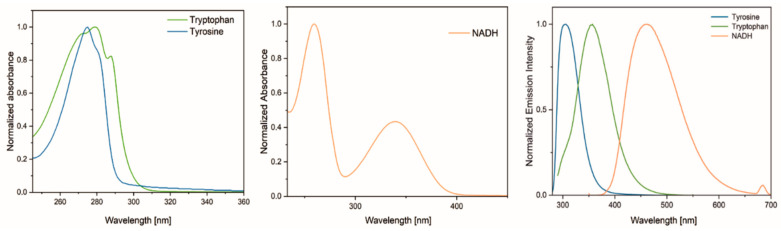
Normalized absorption and emission spectra of reference solutions of tyrosine (blue), tryptophan (green), and the coenzyme NADH (orange).

Following a preliminary assessment of the stability of the spectroscopic properties of tryptophan, an analysis of environmental samples in the form of swab solutions taken from 15 volunteers was initiated. The composition of fingerprints is complex and includes a variety of chemical compounds. These include the amino acids tyrosine and tryptophan, as well as the coenzyme NADH, which are spectroscopically active (Figure 2). The analysis of fingerprint samples using steady-state molecular spectroscopy methods revealed the presence of the aforementioned three components, as well as one additional one. However, this study has focused on the analysis of tryptophan.

**Figure 3:**
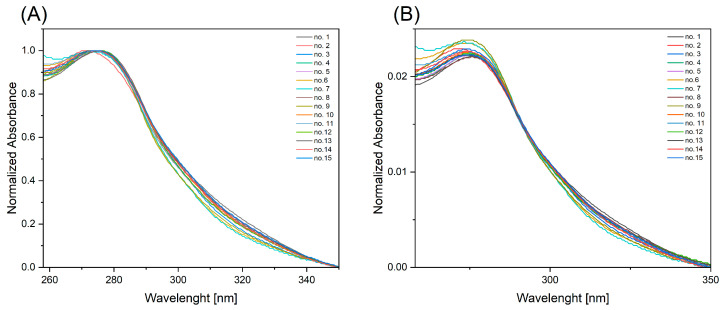
Absorption spectra of solutions obtained from swabbing fingerprint prints left on slides by 15 volunteers: (**A**) absorption spectrum normalized to [0, 1], and (**B**) absorption spectrum normalized to the area.

Absorption spectra of the obtained solutions were measured in the spectral range of 250–800 nm. The absorption spectra of all solutions showed a single band with a maximum located in the range of 274–276 nm. The optical contours of all spectra were similar, with no apparent changes (Figure 3).

**Figure 4:**
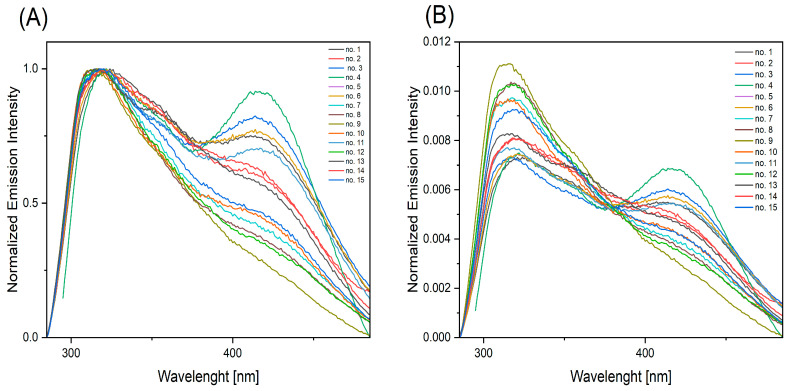
Fluorescence spectra of solutions obtained from swabbing fingerprint prints left on slides by 15 volunteers after excitation with a wavelength of 275 nm: (**A**) fluorescence spectrum normalized to [0, 1], and (**B**) fluorescence spectrum normalized to the area.

Fluorescence spectra were obtained by the excitation of all solutions with two wavelengths: 275 nm (the absorption maximum, as indicated in Figure 3) and 295 nm. An excitation wavelength of 295 nm was selected in order to minimize the excitation of tyrosine and ensure that the resulting emission spectrum predominantly reflects fluorescence from tryptophan. This wavelength is optimal for selectively exciting tryptophan, as tyrosine exhibits negligible absorption at 295 nm, thereby reducing its contribution to the overall fluorescence signal [27]. The fluorescence spectrum excited at a wavelength of 275 nm (Figure 4) was composed of two emission bands between 285 nm and 485 nm for the majority of solutions. The maximum intensity of these bands was observed at wavelengths of approximately 318 nm and 418 nm. Solution No. 9 was the sole exception, exhibiting an absence of a well-developed band at 418 nm. Following the normalization of the spectra to the area, the intersection of the spectra was observed at 380 nm.

**Figure 5:**
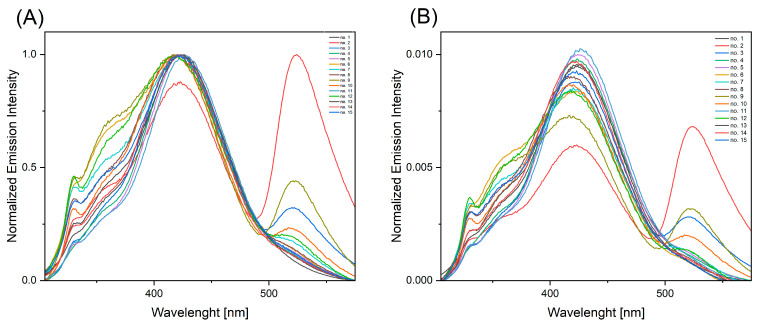
Fluorescence spectra of solutions obtained from swabbing fingerprint prints left on slides by 15 volunteers after excitation with a wavelength of 295 nm: (**A**) fluorescence spectrum normalized to [0, 1], and (**B**) fluorescence spectrum normalized to the area.

Excitation of the solutions with radiation at a wavelength of 295 nm (Figure 5) produced 3 (solutions 1, 4, 5, 11, 14, and 15) or 4 (solutions 2, 3, 6–10, 12, and 13) fluorescence bands falling within the spectral range of 305–575 nm. Maxima were observed at wavelengths of ~330 nm, ~356 nm, ~423 nm, and ~523 nm.

The observed alterations in the optical contour of the analyzed spectra and the discrepancies in the intensity of individual bands can be attributed to the inherent variability in the composition of the sweat–fatty substance.

Excitation of the fluorescence spectra of the solutions with light of a wavelength of 295 nm enabled the differentiation of the aforementioned components of the sweat–fatty substance. The fluorescence spectra of the reference compounds, namely, tryptophan, tyrosine, and NADH, are presented in Figure 2.

**Figure 6:**
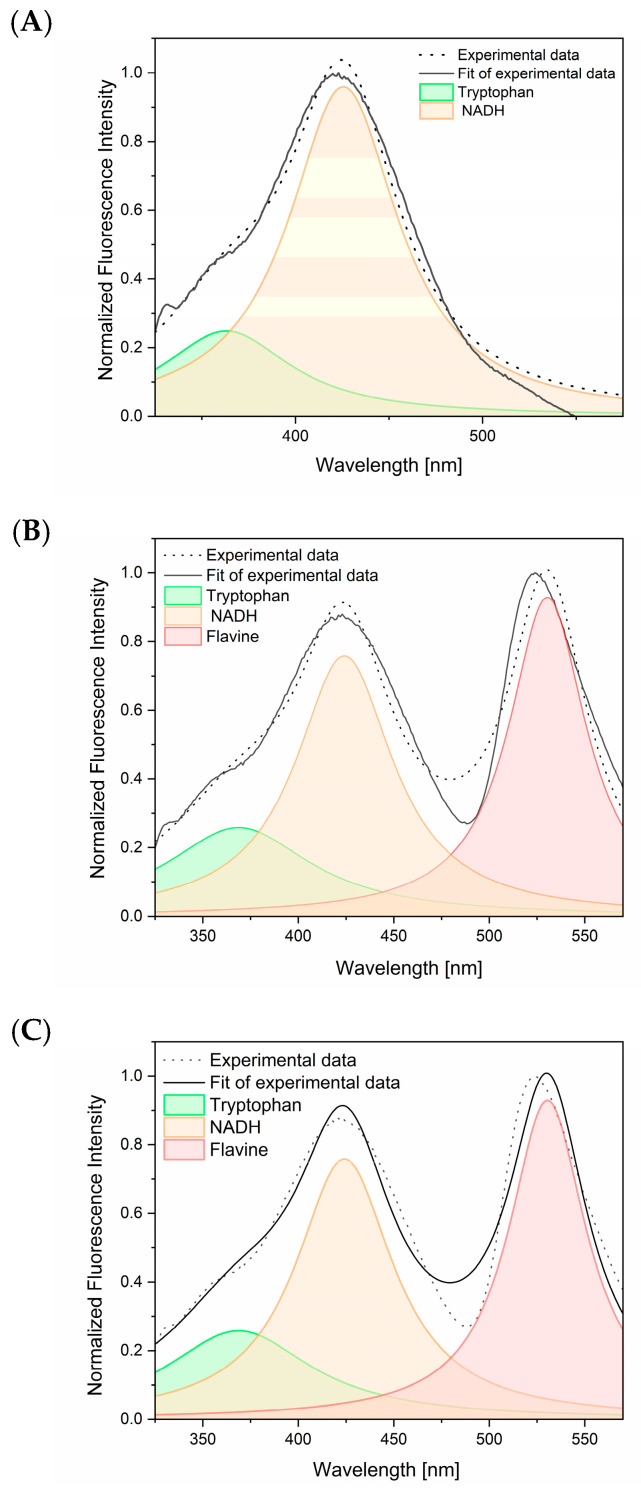
The deconvolution of the three fluorescence spectra of fingerprint samples (Figure 5). (**A**) Sample no. 13, (**B**) sample no. 2, and (**C**) sample no. 3 revealed the apparent matching of the three different fluorescence spectra of tryptophan, NADH, and the fluorescence spectrum of a compound with a fluorescence maximum at 530 nm.

The deconvolution of selected fluorescence spectra revealed the presence of tryptophan and NADH in the analyzed solutions. The deconvolution process was conducted using the actual spectral profiles of the individual component reference compounds (Figure 6). The spectrum of tyrosine was not discernible at a wavelength of 295 nm, which is insufficient for the excitation of this molecule. Furthermore, the latter band discernible in Figure 6B,C may be attributed to flavins, which are also present in the sweat-fatty substance, according to the literature [31].

**Figure 7:**
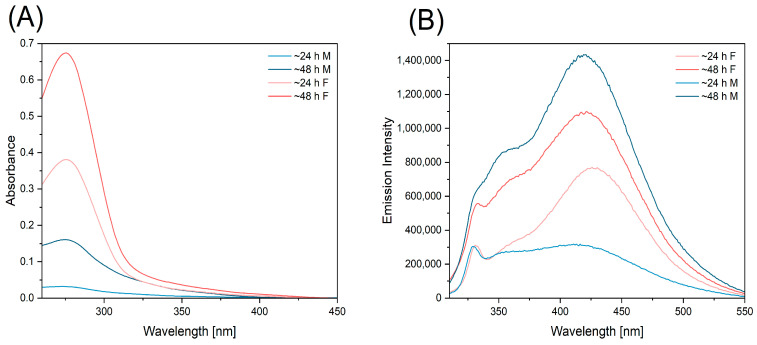
The absorption spectra (**A**) and fluorescence spectra (excitation wavelength 295 nm) (**B**) of samples obtained from a deceased woman (pink) and man (blue) at ~24 and ~48 h post-mortem.

Figure 7A verifies that postmortem secretions exhibit a similar profile of absorption and fluorescence spectra when measured 24 and 48 h after death, in comparison to the sweat–fatty substance collected from living volunteers (these samples served as control samples, confirming the presence of tryptophan in swabs taken from fingerprints). Figure 7B illustrates that the intensity of the fluorescence band maximum for tryptophan (approximately 360 nm) increased significantly with time, which represents a significant contribution to the future design of optical sensors for estimating the time of death. The presented studies clearly demonstrate the potential of tryptophan as a marker for time of death estimations. Nevertheless, additional research will be conducted to examine the impact of other components present in the sweat–fatty substance, with a particular focus on optically active molecules such as tyrosine, NADH, and flavin.

Prior research suggests the potential for tryptophan fluorescence in a sweat–fatty substance; however, a comprehensive spectroscopic characterization of this substance has yet to be conducted. The influence of pH and the presence of other fluorescent components have not been considered. Additionally, the standard location of the fluorescence band maximum has been presented in the literature without further elaboration [31]. In contrast, our results focus on a single biological material and employ a comprehensive spectroscopic approach, underscoring that these findings represent the initial steps in the development of an optical sensor.

## 3. Methodology

### 3.1. Methods

Absorption measurements were performed using a Shimadzu UV-1900i double-beam UV-Vis spectrophotometer (Shimadzu Europa GmbH, Duisburg, Germany) operated in Spectrum Mode. Data acquisition and analysis were conducted using LabSolutions UV-Vis software (version 1.1, Shimadzu Europa GmbH, Duisburg, Germany).

Emission spectra were recorded using a Horiba Jobin Yvon FluoroMax 4 TCSPC spectrofluorometer (HORIBA Europe GmbH, Oberursel, Germany) in excitation mode. Data analysis was carried out using FluorEssence V3 software (version 3.5, HORIBA Europe GmbH, Oberursel, Germany).

### 3.2. Materials

#### 3.2.1. Chemical Standards

Analytical-grade tryptophan, NADH, and tyrosine were sourced from Sigma-Aldrich (Darmstadt, Germany), along with ethanol (96%) from the same supplier. The buffer solutions with pH values of 5, 7, and 8 were prepared using commercially available buffers (EUROCGEM BGD, Tarnów, Poland), consisting of disodium hydrogen phosphate and citric acid. The buffers were mixed in appropriate proportions to achieve the desired pH levels, which were subsequently verified using a pH meter. The pH values were chosen to simulate conditions characteristic of postmortem states of the body.

#### 3.2.2. Sample Preparation

Tryptophan solutions at a concentration of 10^−5^ M were prepared in buffer solutions with pH values of 5, 7, and 8. Samples were equilibrated for 24 h at 2 °C in the absence of light to ensure stability prior to spectral measurements.

#### 3.2.3. Biological Samples from Living Volunteers

Samples were collected from 15 healthy volunteers (10 women and 5 men) aged 20–25 years. Volunteers pressed the index finger of their right hand onto sterile glass slides. Fingerprint residues were then collected using sterile swabs, which were placed in Eppendorf tubes containing 2 mL of a 1% aqueous ethanol solution. The tubes were vortexed for 1 min at 1000 rpm, after which the swabs were removed. Measurements were conducted at 24 h post-collection, with samples stored at 2 °C in the dark. Prior to collection, volunteers refrained from washing their hands for at least two hours to maintain natural skin surface conditions.

#### 3.2.4. Postmortem Sample Collection

**Figure 8:**
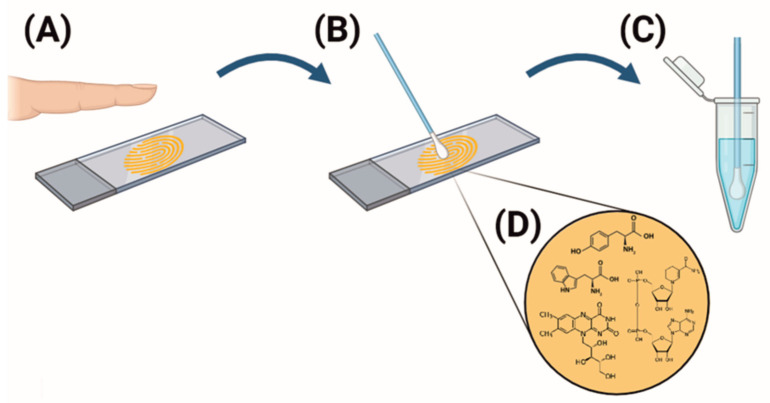
Scheme of the protocol for obtaining research material from volunteers. (**A**) Applying a fingerprint on a slide, (**B**) collecting material with a sterile swab, (**C**) preparing a solution in 1% ethanol, and (**D**) chemical formulas of the compounds present in the sweat–fatty substance.

Samples were collected from two deceased individuals, one male and one female, with the same age range as the living volunteers. The samples were obtained at postmortem intervals of 24 h and 48 h to assess changes in tryptophan stability and concentrations. A sterile swab was used to swipe the skin surface five times at each collection interval. The swabs were processed using the same protocol described for the living volunteers’ samples [Figure 8]. Measurements were taken immediately after the solutions were prepared.

### 3.3. Spectroscopic Measurements

Absorption spectra were recorded for all prepared solutions, including reference standards, samples from living volunteers, and postmortem samples. Measurements were conducted over a range of 200–700 nm. The spectrophotometer was calibrated prior to each session using standard quartz cuvettes and blank solutions. A reference sample containing 1% ethanol was used as a baseline during measurements.

Emission spectra were obtained by exciting the samples at 280 nm (for the reference tryptophan) and at 275 nm and 295 nm (for environmental samples). Measurements were conducted using standardized quartz cuvettes, with a detection range of 290–900 nm. The apparatus was calibrated before the measurements to ensure accuracy.

### 3.4. Mathematical Analysis of Spectra

#### 3.4.1. Normalization to Area

Spectral data were normalized by dividing the intensity values at each wavelength by the total area under the curve. This ensured that comparisons between spectra were independent of sample concentration, emphasizing relative differences in spectral shapes.

#### 3.4.2. Normalization to [0, 1]

Intensities were scaled to a range of [0, 1] by subtracting the minimum intensity and dividing by the range. This normalization minimized baseline offsets and highlighted relative intensity variations across samples.

#### 3.4.3. Peak Deconvolution

Overlapping spectral peaks were resolved using Gaussian fitting techniques and compared with the reference spectra of single-component compounds. The deconvolution analysis identified three spectroscopically active components: tryptophan, NADH, and flavins. Deconvolution was performed using the actual spectral profiles of these components to ensure precise identification.

### 3.5. Ethical Considerations

The study was approved by the Ethics Committee of the University of Gdańsk (Approval No.: 14/2023/WMFiL) for living volunteer samples and by the Bioethics Committee for Scientific Research at the Medical University of Gdańsk (Approval No.: KB/128/2024) for postmortem samples.

Written informed consent was obtained from all participants prior to the commencement of the study.

## 4. Conclusions

The development of a novel, non-invasive approach to estimate the time of death is of paramount importance to the advancement of forensic science. The objective of this study was to validate a hypothesis suggesting the potential use of tryptophan for this purpose. The presence of tryptophan in all body tissues and on the skin surface, the already proven increase in its postmortem concentration, and its susceptibility to degradation under the influence of postmortem processes make it an excellent tool for estimating the time of death.

The research conducted in this study constituted a crucial initial step in exploring the potential of tryptophan as a marker to estimate the time of death. Samples were collected and subjected to a comprehensive analysis, yielding invaluable data that will serve as the foundation for subsequent, more advanced investigations. These data will facilitate a more profound investigation into the correlation between tryptophan levels and the postmortem interval. Subsequent studies will extend the analysis of tryptophan levels to encompass a broader range of samples, across diverse environments, and in conjunction with other biochemical markers. The proposed method offers notable advantages over previously employed techniques, primarily due to its non-invasive nature and the expeditious acquisition of results. The capacity to obtain material for a time of death estimation at the scene of the incident, without the need for an autopsy, improves the accuracy of the subsequent analysis. Furthermore, this approach could help to reduce the impact of prolonged postmortem intervals (PMI) or fluctuating environmental conditions, which often lead to inaccuracies in the results produced by traditional methods. As a result, this comprehensive approach will facilitate the development of a more robust and reliable framework for the estimation of the time since death, which could have significant implications for forensic investigations and the criminal justice system. Moreover, the proposed method is in accordance with the principles of human rights, thereby ensuring that the dignity of human remains is respected.

In conclusion, the findings of this preliminary study represent a substantial advancement in the field of forensic science. The findings establish a foundation for future research that may potentially result in significant advancements in the accurate determination of the postmortem interval.

## Data Availability

The original contributions presented in this study are included in the article. Further inquiries can be directed to the corresponding author(s).
